# Clinical Factors and Composite Biomarkers Associated with Intensive Care Unit-Acquired Weakness: A Retrospective Cohort Study

**DOI:** 10.3390/jcm15208171

**Published:** 2026-10-21

**Authors:** Qiang Zhang, Jie Li, Yi Shu, Qihua Chen, Qiang Lei, Hainan Zhang, Jia Fang

**Affiliations:** 1 Department of Neurology, The Second Xiangya Hospital, Central South University, No. 139 Renmin Middle Road, Furong District, Changsha 410011, China; 238212257@csu.edu.cn (Q.Z.);; 2 Clinical Medical Research Center for Stroke Prevention and Treatment of Hunan Province, Department of Neurology, The Second Xiangya Hospital, Central South University, No. 139 Renmin Middle Road, Furong District, Changsha 410011, China

**Keywords:** intensive care unit-acquired weakness, critical illness, risk factors, sepsis, mechanical ventilation, biomarkers

## Abstract

**Background/Objectives**: Intensive care unit-acquired weakness is a common neuromuscular complication among critically ill patients, but associations of readily available composite biomarkers with this condition remain insufficiently characterized. This study aimed to investigate the clinical characteristics, risk factors, and composite biomarkers associated with intensive care unit-acquired weakness. **Methods**: This single-center retrospective cohort study included 135 critically ill patients admitted to intensive care units between December 2020 and December 2023. Patients were classified into an intensive care unit-acquired weakness group (*n* = 57) and a group without intensive care unit-acquired weakness (*n* = 78) according to Medical Research Council sum score criteria. Clinical characteristics, laboratory parameters, composite biomarkers, and hospitalization outcomes were compared. Multivariable logistic regression and receiver operating characteristic curve analyses were performed. **Results**: Older age, sepsis, hemodialysis, mechanical ventilation, and glucocorticoid use remained associated with intensive care unit-acquired weakness after adjustment for the other covariates in the model. Affected patients had lower albumin and hemoglobin levels and longer intensive care unit and hospital stays. The neutrophil-to-albumin ratio, inflammatory burden index, creatinine-to-albumin ratio, and serum urea-to-creatinine ratio were higher in affected patients. Receiver operating characteristic analysis showed modest discrimination, with areas under the curve ranging from 0.602 to 0.665. **Conclusions**: Intensive care unit-acquired weakness was associated with clinical and treatment-related factors, hypoalbuminemia, prolonged hospitalization, and elevated composite biomarkers. The composite biomarkers showed only modest discrimination, and their incremental values beyond clinical assessment and clinical utility remain unestablished. Residual confounding by illness severity and nutritional exposure cannot be excluded.

## 1. Introduction

Intensive care unit-acquired weakness (ICUAW) is a severe neuromuscular disorder that develops in critically ill patients during intensive care. It typically causes symmetric weakness of the respiratory and limb muscles, often with greater involvement of proximal limb muscles. Persistent physical disability may continue after hospital discharge and impair quality of life [1]. The pathophysiological mechanisms underlying ICUAW remain incompletely understood and may involve multiple interacting processes, including inflammation, microcirculatory dysfunction, altered nutritional metabolism, and neuromuscular impairment [2]. ICUAW occurs in approximately 40% of critically ill patients with multiple organ failure, prolonged mechanical ventilation, or sepsis [3]. ICUAW is strongly associated with difficult weaning from mechanical ventilation, prolonged intensive care unit (ICU) and hospital stays, increased healthcare costs, and higher in-hospital mortality [4]. Clinically, ICUAW is defined as newly acquired muscle weakness in critically ill patients that cannot be attributed to any cause other than critical illness [5]. Because no specific treatment is currently available, the prevention and management of ICUAW rely largely on the early identification and modification of risk factors.

Several risk factors for ICUAW have been identified, including sepsis, prolonged mechanical ventilation, a higher Acute Physiology and Chronic Health Evaluation II (APACHE II) score, hyperlactatemia, prolonged ICU stay, and sedative use [6,7]. Other studies have reported female sex, an APACHE II score > 12, a Sequential Organ Failure Assessment (SOFA) score > 7, hyperlactatemia, diabetes mellitus, electrolyte disturbances, systemic inflammatory response syndrome, sepsis, and multiple organ failure as potential or independent risk factors for ICUAW [8]. However, other studies have reported no significant association between female sex and ICUAW, and the potential mechanisms underlying this relationship remain unclear [9]. These inconsistent findings suggest that the pathogenesis of ICUAW and the predictive value of its associated risk factors remain incompletely understood.

Current evidence supports early interventions for critically ill patients, including rehabilitation; implementation of the Assess, prevent, and manage pain; Both spontaneous awakening and breathing trials; Choice of analgesia and sedation; Delirium assessment, prevention, and management; Early mobility and exercise; and Family engagement and empowerment (ABCDEF) bundle; and individualized nutritional support as strategies to improve ICUAW-related outcomes [10,11]. The successful implementation of these interventions depends on timely recognition of ICUAW. However, its diagnosis still relies primarily on clinical assessment of muscle strength, and sensitive and specific biomarkers remain lacking. Moreover, differences in the availability of intervention strategies across healthcare settings further complicate early recognition and management [12]. Biomarkers are objective and quantifiable indicators of physiological or pathological processes that may facilitate early diagnosis, disease monitoring, and prognostic assessment. Therefore, there is an urgent need for noninvasive, readily available, and objective biomarkers to facilitate the early diagnosis and management of ICUAW [13]. Systemic inflammation is a recognized etiologic criterion for malnutrition and may reduce nutritional intake, alter metabolism, increase energy expenditure, and promote muscle catabolism and atrophy [14]. The magnitude of the inflammatory response can be assessed using peripheral blood cell counts and circulating inflammatory biomarkers, including neutrophil and lymphocyte counts and C-reactive protein levels [15]. The creatinine-to-albumin ratio (CAR) has emerged as a composite biomarker with potential pathophysiological relevance to ICUAW. By integrating serum creatinine, an indicator of renal function and muscle metabolism, with serum albumin, which is affected by inflammation and fluid shifts, CAR may capture overlapping physiological disturbances and has demonstrated prognostic value in critically ill populations [16]. In addition, the serum urea-to-creatinine ratio (SUCR) may reflect altered protein metabolism, including endogenous catabolism and exogenous protein delivery, and is also influenced by renal function [17,18].

Despite increasing recognition of ICUAW, it remains unclear which readily available measurements are associated with its occurrence. Composite biomarkers integrating inflammatory, metabolic, and renal processes have demonstrated prognostic value in critically ill populations; however, their associations with ICUAW have not been systematically investigated.

Therefore, this study aimed to characterize the clinical features and factors associated with ICUAW and to assess the apparent discriminatory ability of four composite biomarkers, including the neutrophil-to-albumin ratio (NAR), inflammatory burden index (IBI), CAR, and SUCR.

## 2. Materials and Methods

### 2.1. Study Design

In this retrospective study, patients were classified according to the Medical Research Council sum score (MRC-ss) into the ICUAW group (MRC-ss < 48) or the non-ICUAW group (MRC-ss ≥ 48) [19]. Clinical characteristics, potential risk factors, and biomarker levels were subsequently compared between the two groups.

This study was reported in accordance with the REporting of studies Conducted using Observational Routinely collected health Data (RECORD) statement [20].

The study protocol was approved by the Ethics Committee of the Second Xiangya Hospital of Central South University (approval number: LYF20260198).

### 2.2. Setting and Participants

This study included 135 patients admitted to the intensive care units at the Second Xiangya Hospital of Central South University between December 2020 and December 2023. The participating units included the neurological ICU, general ICU, respiratory and critical care ICU, and emergency ICU. Patients were classified into the ICUAW group (*n* = 57) and the non-ICUAW group (*n* = 78) according to the presence or absence of ICUAW. Data on potential risk factors, clinical characteristics, and laboratory and ancillary examination findings were extracted from the medical records.

### 2.3. Eligibility and Diagnostic Criteria

The inclusion criteria for the ICUAW group were as follows: age ≥ 18 years; complete clinical data for the assessment of relevant variables; an MRC-ss ≥ 48 at ICU admission; subsequent development of ICUAW according to established clinical diagnostic criteria; and sufficient consciousness and cooperation to undergo muscle strength assessment.

The inclusion criteria for the non-ICUAW group were as follows: age ≥ 18 years; complete clinical data for the assessment of relevant variables; an MRC-ss ≥ 48 at ICU admission; no subsequent development of ICUAW during the ICU stay; and sufficient consciousness and cooperation to undergo muscle strength assessment.

The exclusion criteria were as follows: pre-existing neuromuscular disorders associated with muscle weakness at ICU admission, such as amyotrophic lateral sclerosis, polymyositis, or Guillain–Barré syndrome; an ICU stay of ≤48 h; an MRC-ss < 48 at ICU admission; withdrawal from active treatment; or incomplete clinical data. Patients who were deeply sedated, comatose, or otherwise unable to follow commands at the required MRC-ss assessment time points were excluded because a reliable muscle strength assessment could not be obtained.

ICUAW was diagnosed in conscious and cooperative patients using the MRC-ss. The score evaluates six bilateral muscle movements: shoulder abduction, elbow flexion, wrist extension, hip flexion, knee extension, and ankle dorsiflexion. Muscle strength was assessed at least twice at 24 h intervals. An MRC-ss < 48 was considered diagnostic of ICUAW.

### 2.4. Data Collection

Clinical data were retrospectively extracted from the medical records of patients in both groups. The variables collected were categorized as follows:

Demographic variables included age and sex.

Pre-existing comorbidities included diabetes mellitus, hypertension, and other relevant medical conditions.

Clinical and treatment-related variables included the primary diagnosis; sepsis; septic shock; parenteral nutrition; vasoactive agent use; hemodialysis; mechanical ventilation; mechanical ventilation for >48 h; tracheostomy; and exposure to glucocorticoids, sedatives, neuromuscular blocking agents, and aminoglycoside antibiotics. Parenteral nutrition was recorded as a binary variable, but total caloric and protein intake from all routes was not quantified in the analytic dataset.

Laboratory variables included serum albumin levels, white blood cell count, C-reactive protein (CRP), procalcitonin (PCT), erythrocyte sedimentation rate (ESR), lactate, hemoglobin, liver and renal function parameters, and electrolyte levels.

Hospitalization outcomes included ICU length of stay and total hospital length of stay.

The composite biomarkers evaluated were the neutrophil-to-albumin ratio (NAR); inflammatory burden index (IBI), calculated as C-reactive protein (CRP) × neutrophil count/lymphocyte count; creatinine-to-albumin ratio (CAR); and serum urea-to-creatinine ratio (SUCR).

NAR was calculated as the absolute neutrophil count (×10^9^ cells/L) divided by the serum albumin concentration (g/L), giving values in 10^9^ cells/g. The recorded numeric values are the direct quotient of these two measurements; they were analyzed on the original ratio scale without logarithmic transformation or other rescaling.

The laboratory values used to calculate the composite biomarkers were generally obtained from the first available blood sample after hospital admission, before ICU transfer when patients entered the ICU later; for patients admitted directly to the ICU, the first sample after ICU admission was used. The components of each index were obtained from the same sampling episode; measurements were not selected specifically at the time of ICUAW diagnosis. Because exact blood-sampling dates/times and dates of first ICUAW diagnosis were incomplete for some patients, we could not verify that these measurements preceded ICUAW onset or diagnosis in every case.

### 2.5. Statistical Analysis

IBM SPSS Statistics for Windows (version 26.0.1; IBM Corp., Armonk, NY, USA) was used for the primary analyses [21]. Continuous variables were examined for normality. Group differences were tested with the independent-samples *t*-test for normally distributed values, reported as mean ± standard deviation, and with the Mann–Whitney *U* test for non-normally distributed values, reported as median (interquartile range). Categorical data were summarized as *n* (%) and compared using the chi-square test. Age, sepsis, hemodialysis, mechanical ventilation, and glucocorticoid use were selected before model fitting on the basis of clinical relevance and prior evidence; their associations with ICUAW were estimated by multivariable logistic regression. Variance inflation factors (VIFs) were calculated for the five covariates. During initial chart review, APACHE II and SOFA scores were absent from many patient records; these variables were therefore not systematically abstracted and were not entered into the full-cohort model. Separate receiver operating characteristic (ROC) curves for NAR, IBI, CAR, and SUCR were used to describe unadjusted discrimination between patients with and without ICUAW. For each biomarker, we calculated the area under the curve (AUC) and its 95% confidence interval (CI), then identified the cohort-specific cutoff maximizing the Youden index. All tests were two-sided, with *p* < 0.05 considered statistically significant.

To assess the internal stability of these cutoffs, we performed 1000 patient-level stratified bootstrap resamples, sampling patients with and without ICUAW separately with replacement to preserve group sizes. In each resample, the cutoff maximizing the Youden index was re-estimated for each biomarker; when multiple cutoffs tied, the lowest was selected. We summarized the bootstrap distributions of cutoffs and AUCs using medians and empirical 2.5th and 97.5th percentiles. Each bootstrap-derived cutoff was applied to patients not selected in that resample (out-of-bag) to estimate sensitivity and specificity. The analysis included the same 135 patients as the primary ROC analyses. Bootstrap calculations were implemented in Python 3.12.14 using NumPy 2.3.5.

### 2.6. Use of Generative Artificial Intelligence

OpenAI Codex was used solely to assist with English-language editing and manuscript formatting. It was not used for study design, data collection, data analysis, data interpretation, or figure generation. All outputs were reviewed and edited by the authors, who take full responsibility for the content of the article.

## 3. Results

### 3.1. Demographic Characteristics and Comorbidities

The mean age was significantly higher in the ICUAW group than in the non-ICUAW group (61.04 ± 15.92 vs. 51.94 ± 18.68 years; *t* = −2.972, *p* = 0.004). The proportion of patients aged >60 years was significantly higher in the ICUAW group than in the non-ICUAW group (56.14% vs. 38.46%; χ^2^ = 4.145, *p* = 0.042). The proportion of female patients was higher in the ICUAW group than in the non-ICUAW group, although the difference was not statistically significant (45.61% vs. 32.05%; χ^2^ = 2.577, *p* = 0.108) (Table 1).

No significant between-group differences were observed in the prevalence of diabetes mellitus (33.33% vs. 29.49%; χ^2^ = 0.227, *p* = 0.634) or hypertension (45.61% vs. 37.18%; χ^2^ = 0.997, *p* = 0.318) (Table 1).

### 3.2. Clinical and Treatment-Related Characteristics

The most common primary diagnoses in the ICUAW group were sepsis (82.46%) and severe pneumonia (11.34%). Sepsis, septic shock, parenteral nutrition, vasoactive agent use, hemodialysis, mechanical ventilation, tracheostomy, glucocorticoid use, sedative use, neuromuscular blocking agent use, mechanical ventilation for >48 h, and aminoglycoside antibiotic use were all significantly more frequent in the ICUAW group than in the non-ICUAW group (all *p* < 0.05) (Table 1).

### 3.3. Length of Stay

Both ICU and total hospital lengths of stay were significantly longer in the ICUAW group than in the non-ICUAW group (both *p* < 0.001). Because both variables were non-normally distributed, they are presented as medians with interquartile ranges. The median ICU length of stay was 40.00 (21.50–45.00) days in the ICUAW group, compared with 8.50 (5.00–12.00) days in the non-ICUAW group (*Z* = 8.246, *p* < 0.001). The median total hospital length of stay was 40.00 (25.50–49.00) days in the ICUAW group, compared with 8.50 (5.00–12.00) days in the non-ICUAW group (*Z* = 8.400, *p* < 0.001) (Table 2).

### 3.4. Laboratory Parameters

The prevalence of electrolyte disturbances was significantly higher in the ICUAW group than in the non-ICUAW group (78.95% vs. 56.41%; χ^2^ = 7.446, *p* = 0.006). The prevalence of renal dysfunction was also significantly higher in the ICUAW group than in the non-ICUAW group (50.88% vs. 19.23%; χ^2^ = 15.013, *p* < 0.001). In contrast, the prevalence of liver dysfunction did not differ significantly between the ICUAW and non-ICUAW groups (49.12% vs. 53.85%; χ^2^ = 0.156, *p* = 0.693) (Table 3).

### 3.5. Multivariable Logistic Regression Analysis

The presence of ICUAW was entered as the dependent variable and coded as 0 (absence of ICUAW) or 1 (presence of ICUAW). Variables showing statistically significant associations in univariable analyses were considered candidate predictors, together with clinically relevant variables selected a priori based on prior evidence. To limit model complexity in the setting of 57 ICUAW events, five clinically relevant variables previously associated with ICUAW were included in the multivariable logistic regression model: age, sepsis, hemodialysis, mechanical ventilation, and glucocorticoid use. Age, sepsis, hemodialysis, mechanical ventilation, and glucocorticoid use remained associated with ICUAW in the five-variable model (all *p* < 0.05). The VIFs were 1.076 for age, 1.537 for sepsis, 1.201 for hemodialysis, 1.817 for mechanical ventilation, and 1.943 for glucocorticoid use (Table 4), providing no evidence of substantial multicollinearity. Despite large point estimates for hemodialysis (OR 26.023, 95% CI 4.047–167.308) and glucocorticoid use (OR 21.978, 95% CI 4.049–119.292), their wide confidence intervals indicate marked imprecision. APACHE II and SOFA scores were not systematically abstracted, precluding full-cohort adjustment for baseline illness severity.

### 3.6. Composite Biomarkers

The levels of all four composite biomarkers were significantly lower in the non-ICUAW group than in the ICUAW group: NAR (*Z* = −2.873, *p* = 0.004), IBI (*Z* = −2.014, *p* = 0.044), CAR (*Z* = −2.713, *p* = 0.007), and SUCR (*Z* = −3.270, *p* = 0.001) (Table 5).

### 3.7. ROC Curve Analysis of Composite Biomarkers

**Figure 1:**
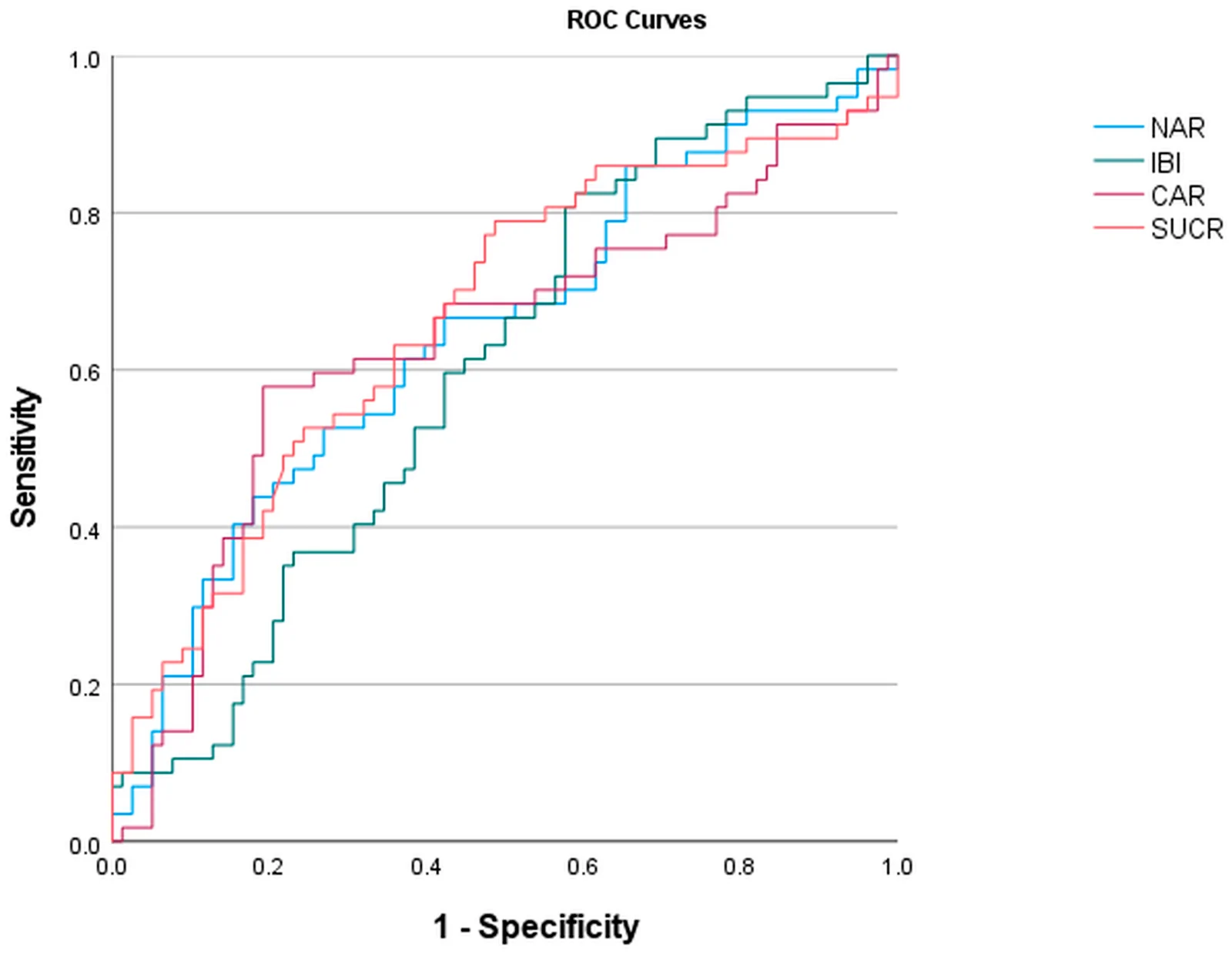
Receiver operating characteristic curves of the composite biomarkers for discriminating between patients with and without intensive care unit-acquired weakness. Abbreviations: CAR, creatinine-to-albumin ratio; IBI, inflammatory burden index; NAR, neutrophil-to-albumin ratio; ROC, receiver operating characteristic; SUCR, serum urea-to-creatinine ratio.

ROC curve analysis demonstrated statistically significant but modest discriminatory ability for all four composite biomarkers, as reflected by their AUC values (Figure 1). SUCR yielded the highest AUC (AUC = 0.665, 95% CI 0.570–0.760, *p* = 0.001), followed by NAR (AUC = 0.645, 95% CI 0.550–0.740, *p* = 0.004), CAR (AUC = 0.637, 95% CI 0.538–0.736, *p* = 0.007), and IBI (AUC = 0.602, 95% CI 0.506–0.697, *p* = 0.044). The corresponding sample-derived cutoff values, sensitivities, specificities, and Youden indices are presented in Table 6. All AUC values were <0.70, indicating modest discriminatory ability. These analyses assessed each biomarker individually; incremental performance after addition to a clinical prediction model was not evaluated. These AUCs describe discrimination between ICUAW groups in this retrospective cohort and should not be interpreted as prospective predictions of incident ICUAW.

Across 1000 bootstrap resamples, median cutoffs (empirical 2.5th–97.5th percentiles) were 0.354 (0.182–0.462) for NAR, 388.226 (137.404–2293.747) for IBI, 4.267 (3.377–4.450) for CAR, and 0.068 (0.058–0.115) for SUCR (Supplementary Table S1). The corresponding bootstrap AUC percentile intervals were 0.544–0.741, 0.498–0.697, 0.543–0.742, and 0.566–0.756, respectively. Out-of-bag evaluation yielded lower mean Youden indices than the apparent estimates for each cutoff-selection procedure. The especially wide cutoff distribution for IBI and variable out-of-bag operating characteristics indicate that these thresholds are exploratory.

## 4. Discussion

This retrospective cohort study identified several clinical and treatment-related factors, laboratory parameters, and composite biomarkers associated with ICUAW. These findings are discussed below in terms of their potential clinical implications and biological plausibility.

### 4.1. Demographic Characteristics and Comorbidities

Older age remained associated with ICUAW in the five-variable model. This finding is consistent with previous studies and may reflect age-related declines in physiological reserve, immune function, and muscle mass, as well as reduced tolerance to the stress of critical illness. During their ICU stay, older critically ill patients may be more susceptible to systemic inflammation, nutritional deficiencies, immobilization, and other adverse factors that contribute to neuromuscular dysfunction and increase the risk of ICUAW.

No significant between-group differences were observed in the proportion of female patients or the prevalence of diabetes mellitus and hypertension (all *p* > 0.05). These findings suggest that sex, diabetes mellitus, and hypertension were not significantly associated with ICUAW in this cohort. Our finding differs from a meta-analysis that identified female sex as a risk factor for ICUAW [22]. However, it is consistent with a sepsis cohort in which sex was not identified as an independent risk factor [23]. These discrepancies may reflect differences in sample size, illness severity, and the degree of comorbidity control across study populations. The association between sex and ICUAW and the mechanisms underlying this relationship remain unclear and warrant further investigation in larger multicenter studies.

### 4.2. Clinical and Treatment-Related Factors

Sepsis is among the most consistently reported risk factors for ICUAW. This association may be mediated, at least in part, by sepsis-induced systemic inflammation. During sepsis, high levels of inflammatory mediators, including tumor necrosis factor-α and interleukin-6, are released. These mediators may impair neuromuscular junction function, inhibit muscle protein synthesis, promote muscle catabolism, and induce microcirculatory dysfunction, resulting in tissue ischemia and hypoxia and ultimately contributing to muscle weakness [24,25,26]. Severe pneumonia may precipitate sepsis and systemic inflammation, thereby increasing exposure to these catabolic pathways [27]. Severe acute respiratory syndrome coronavirus 2 infection may also involve skeletal muscle through direct or inflammation-associated mechanisms, resulting in myalgia, creatine kinase elevation, or myositis [28].

The observed association between hemodialysis and ICUAW should not be interpreted as evidence of a direct causal relationship. Rather, the need for hemodialysis may indicate more severe renal dysfunction, a greater systemic inflammatory burden, and more extensive multiple organ dysfunction, all of which may increase susceptibility to ICUAW [8,29,30].

Acute kidney injury and kidney replacement therapy may contribute to muscle wasting through altered amino acid and protein metabolism, inflammatory signaling, and treatment-related loss of nutrients. These mechanisms may increase susceptibility to ICUAW [31].

Glucocorticoids are commonly used in the ICU to manage inflammation and shock. However, prolonged or high-dose glucocorticoid exposure may promote muscle catabolism and impair neuromuscular function. A prospective multicenter study identified corticosteroid exposure as an independent risk factor for ICU-acquired paresis [32].

Patients undergoing mechanical ventilation are frequently exposed to prolonged immobilization and sedative medications. Prolonged immobilization may lead to disuse muscle atrophy and impaired neuromuscular function. In addition, neuromuscular blocking agents may impair neuromuscular transmission and prolong mechanical ventilation, potentially exacerbating neuromuscular injury [33,34].

Because APACHE II and SOFA scores were not systematically collected, the full-cohort model could not directly adjust for baseline illness severity using these scores. Severity may be related to sepsis, hemodialysis, mechanical ventilation, glucocorticoid use, and ICUAW. The observed associations may therefore partly reflect residual confounding or treatment indication. These estimates are conditional on the included covariates and should not be interpreted as independent causal effects. The direction and magnitude of any residual confounding cannot be determined from these data. The larger adjusted OR point estimates for hemodialysis and glucocorticoid use should not be interpreted as evidence that these are the strongest risk factors.

### 4.3. Laboratory Parameters and Hospitalization Outcomes

Hypoalbuminemia in critical illness may reflect inflammation and fluid shifts rather than inadequate nutritional intake. A retrospective study of mechanically ventilated critically ill patients found lower albumin levels in patients with ICUAW and identified low serum albumin as a potential ICUAW risk factor [35]. Accordingly, serum albumin should be interpreted as a nonspecific associated marker rather than a causal determinant of ICUAW or a direct measure of nutritional intake [36].

The prolonged ICU and hospital stays observed among patients with ICUAW may be attributable to difficult weaning from mechanical ventilation, limb weakness, delayed mobilization, and the need for extended monitoring, treatment, and rehabilitation. Prolonged hospitalization may increase healthcare costs and impose substantial burdens on patients and their families, underscoring the clinical importance of early recognition and prevention of ICUAW.

This study evaluated four composite biomarkers, namely NAR, IBI, CAR, and SUCR, which integrate information on systemic inflammation, protein metabolism, and renal function. Although statistically significant, the individual AUCs indicated only modest discrimination. These results do not establish whether the biomarkers improve clinical risk assessment or support management decisions.

NAR combines neutrophil count with serum albumin [37]. IBI combines CRP with neutrophil and lymphocyte counts [38,39], CAR combines serum creatinine with albumin [40], and SUCR has been proposed as a marker of catabolism [41]. These indices reflect overlapping inflammatory, renal, and metabolic processes rather than specific nutritional states. In critical illness, serum albumin is affected by inflammation and fluid shifts and should not be interpreted as a direct measure of caloric or protein intake [36]. SUCR may also vary with exogenous protein delivery and renal function, in addition to endogenous catabolism [18]. Because total caloric and protein intake was not quantified, we could not separate these influences or establish a nutritional mechanism for the observed biomarker associations.

Although these biomarkers can be calculated from routinely available laboratory measurements, the observed AUCs of 0.602–0.665 and the sensitivity–specificity trade-offs at data-derived cutoffs do not support their use as standalone tests to diagnose ICUAW or guide interventions. Statistical significance relative to chance does not establish added value beyond clinical information. Their potential clinical utility requires evaluation in prospectively collected cohorts with prespecified prediction times, validation, calibration, and assessment of clinical net benefit.

### 4.4. Strengths and Limitations

This study has several notable strengths. First, this study systematically evaluated clinical characteristics, treatment-related factors, laboratory parameters, and composite biomarkers associated with ICUAW in a real-world ICU cohort. Second, this study evaluated readily available biomarkers, including NAR, IBI, CAR, and SUCR, which integrate inflammatory, renal, and metabolic laboratory signals and warrant further evaluation as candidate biomarkers. Third, ROC analysis provided exploratory estimates of individual discrimination and data-derived cutoffs.

Nevertheless, this study has several limitations. First, the single-center retrospective design and relatively small sample size may have introduced selection bias and residual confounding. The limited number of ICUAW events also constrained the number of variables that could be included in the multivariable model, precluding full adjustment for some potentially relevant confounders. Although the VIFs did not indicate substantial multicollinearity, the limited sample size may have reduced the precision and stability of the multivariable estimates. The large odds ratios with wide confidence intervals should therefore be interpreted cautiously.

Second, these biomarkers showed only modest discriminatory performance. Although we assessed the sample-derived Youden cutoffs in 1000 bootstrap resamples, several cutoffs varied widely, particularly IBI. The bootstrap AUC percentile interval for IBI (0.498–0.697) included 0.5, indicating substantial sampling variability in its estimated discrimination. Out-of-bag operating characteristics were also less favorable than apparent estimates. Internal resampling cannot establish performance in other settings; these thresholds remain exploratory pending external validation. Temporal changes in these biomarkers during the ICU stay were also not assessed.

Third, preliminary chart review showed that APACHE II and SOFA scores were absent from many records, so these variables were not systematically abstracted for the full cohort. Their exact missingness percentages cannot be calculated from the study dataset, and they could not be included in the full-cohort model. Baseline illness severity may influence both the need for organ-support therapies and ICUAW; therefore, residual confounding by severity and treatment indication remains possible. The direction and magnitude of this bias cannot be determined, and the VIF analysis does not address omitted-variable confounding. We did not assess whether adding the biomarkers to a standard severity-score model improved the AUC or net reclassification. Their incremental value beyond established clinical assessment remains unknown.

Because MRC-ss assessment requires patient cooperation, patients who were deeply sedated, comatose, or otherwise unable to follow commands at the required assessment points were not included in the analytic cohort. These patients may have had greater illness severity and more frequent exposure to prolonged sedation or neuromuscular blocking agents. Their exclusion may have affected both the observed proportion of ICUAW and the estimated associations with clinical and treatment-related factors; the magnitude and direction of this bias cannot be determined from the available data. Therefore, our findings should be interpreted as applying to MRC-assessable patients rather than to all ICU admissions.

Because exact blood-sampling times and dates of first ICUAW diagnosis were incomplete for some patients, we could not verify that biomarker measurement preceded ICUAW in every case. Reverse causality and other time-related bias therefore cannot be fully excluded, and these biomarkers should be interpreted as associated with ICUAW rather than as validated early predictors.

Quantitative total caloric and protein intake was not available; a binary record of parenteral nutrition cannot represent actual nutritional exposure. We could not determine whether presampling intake influenced the baseline biomarkers or whether subsequent intake affected ICUAW. The contribution of nutrition to the observed associations therefore remains uncertain. Long-term functional outcomes after hospital discharge were also not assessed. Future prospective studies with complete baseline severity scores and time-aligned nutritional and laboratory data are warranted.

## 5. Conclusions

Based on this retrospective analysis of 135 critically ill patients, which incorporated clinical data, laboratory parameters, and composite biomarkers, several conclusions can be drawn. ICUAW was common among critically ill patients and was associated with older age, treatment-related factors, systemic inflammation, protein metabolism, and renal function. ICUAW was also associated with significantly longer ICU and overall hospital stays, suggesting an association with poorer clinical outcomes.

Older age, sepsis, the need for hemodialysis, mechanical ventilation, and glucocorticoid use remained associated with ICUAW after adjustment for the other measured covariates; residual confounding by illness severity cannot be excluded.

Patients with ICUAW had significantly lower serum albumin levels, which may reflect inflammation and other illness-related changes rather than inadequate nutrient intake alone; serum albumin should therefore be considered a nonspecific associated marker. In addition, four composite biomarkers, namely NAR, IBI, CAR, and SUCR, were significantly associated with ICUAW. These biomarkers should be considered exploratory findings rather than validated tools for clinical decision-making; their incremental value beyond clinical assessment remains unknown.

## Figures and Tables

**Table 1:** Demographic, comorbidity, and treatment-related characteristics.

Variable	Non-ICUAW Group (*n* = 78)	ICUAW Group (*n* = 57)	*t*/χ^2^	*p* Value
Female, *n* (%)	25 (32.05)	26 (45.61)	2.577	0.108
Age, years, mean ± standard deviation	51.94 ± 18.68	61.04 ± 15.92	−2.972	0.004
Age group, years				
≤60	48 (61.54)	25 (43.86)		
>60	30 (38.46)	32 (56.14)	4.145	0.042
Sepsis, *n* (%)	15 (19.23)	47 (82.46)	53.012	<0.001
Septic shock, *n* (%)	10 (12.82)	40 (70.18)	46.457	<0.001
Hypertension, *n* (%)	29 (37.18)	26 (45.61)	0.997	0.318
Diabetes mellitus, *n* (%)	23 (29.49)	19 (33.33)	0.227	0.634
Parenteral nutrition, *n* (%)	39 (50.00)	49 (85.96)	18.771	<0.001
Vasoactive agent use, *n* (%)	18 (23.08)	47 (82.46)	46.512	<0.001
Hemodialysis, *n* (%)	10 (12.82)	26 (45.61)	18.111	<0.001
Mechanical ventilation, *n* (%)	15 (19.23)	47 (82.46)	53.012	<0.001
Tracheotomy, *n* (%)	4 (5.13)	26 (45.61)	30.796	<0.001
Glucocorticoid use, *n* (%)	13 (16.67)	49 (85.96)	63.684	<0.001
Sedative use, *n* (%)	35 (44.87)	53 (92.98)	33.590	<0.001
Neuromuscular blocking agent use, *n* (%)	10 (12.82)	35 (61.40)	34.980	<0.001
Duration of mechanical ventilation (h)				
≤48	64 (82.05)	28 (49.12)		
>48	14 (17.95)	29 (50.88)	16.660	<0.001
Aminoglycoside antibiotic use, *n* (%)	0 (0.00)	9 (15.79)	10.780	0.001

Note: Data are presented as *n* (%) unless otherwise indicated. Abbreviations: ICUAW, intensive care unit-acquired weakness.

**Table 2:** Laboratory parameters and length of stay.

Variable	Non-ICUAW Group (*n* = 78)	ICUAW Group (*n* = 57)	U	Z	*p* Value
White blood cell count (×10^9^/L)	10.46 (6.72–14.03)	11.22 (8.10–15.60)	2476.5	1.129	0.259
Total hospital length of stay (days)	8.50 (5.00–12.00)	40.00 (25.50–49.00)	4107	8.400	<0.001
ICU length of stay (days)	8.50 (5.00–12.00)	40.00 (21.50–45.00)	4072.5	8.246	<0.001
Albumin (g/L)	34.4 (29.9–38.3)	27.9 (25.5–31.05)	939.5	−5.718	<0.001
C-reactive protein (mg/L)	79.20 (29.20–182.60)	113.00 (64.90–179.50)	2475	1.553	0.120
Procalcitonin (ng/mL)	0.68 (0.13–3.93)	1.20 (0.25–7.57)	2414	1.758	0.079
Serum calcium (mmol/L)	1.95 (1.85–2.05)	1.91 (1.79–2.04)	2006	−0.967	0.334
Lactate (mmol/L)	1.40 (0.90–2.40)	1.30 (0.90–2.76)	2285	0.276	0.782
Erythrocyte sedimentation rate (mm/h)	45.0 (14.5–72.5)	50.0 (23.0–90.0)	370.5	0.605	0.545
Hemoglobin (g/L)	120.0 (95.0–131.0)	95.0 (74.5–108.0)	1179.5	−4.650	<0.001

Note: Data are presented as median (interquartile range). Abbreviations: ICU, intensive care unit; ICUAW, intensive care unit-acquired weakness.

**Table 3:** Categorical laboratory abnormalities.

Variable	Non-ICUAW Group (*n* = 78)	ICUAW Group (*n* = 57)	χ^2^	*p* Value
Electrolyte disturbances, *n* (%)	44 (56.41)	45 (78.95)	7.446	0.006
Liver dysfunction, *n* (%)	42 (53.85)	28 (49.12)	0.156	0.693
Renal dysfunction, *n* (%)	15 (19.23)	29 (50.88)	15.013	<0.001

Note: Data are presented as *n* (%). Abbreviations: ICUAW, intensive care unit-acquired weakness.

**Table 4:** Multivariable logistic regression analysis of factors associated with ICUAW.

Variable	B	SE	Wald χ^2^	*p* Value	OR	95% CI	VIF
Age	0.076	0.0231	10.595	0.001	1.079	1.031–1.129	1.076
Sepsis	2.287	0.7061	10.483	0.001	9.842	2.466–39.288	1.537
Hemodialysis	3.259	0.949	11.782	<0.001	26.023	4.047–167.308	1.201
Mechanical ventilation	1.598	0.744	4.613	0.032	4.944	1.150–21.260	1.817
Glucocorticoid use	3.090	0.863	12.819	<0.001	21.978	4.049–119.292	1.943

Note: Variables were entered simultaneously into the multivariable logistic regression model. VIF, variance inflation factor. Abbreviations: B, regression coefficient; CI, confidence interval; ICUAW, intensive care unit-acquired weakness; OR, odds ratio; SE, standard error.

**Table 5:** Composite biomarker levels.

Variable	Non-ICUAW Group (*n* = 78)	ICUAW Group (*n* = 57)	U	Z	*p* Value
NAR (10^9^ cells/g)	0.257 (0.140–0.374)	0.361 (0.203–0.507)	1578	−2.873	0.004
IBI (mg/L)	814.68 (149.79–2101.55)	1310.83 (481.33–2922.03)	1771	−2.014	0.044
CAR (mmol/g)	2.604 (1.927–3.736)	4.714 (2.163–10.458)	1614	−2.713	0.007
SUCR	0.064 (0.050–0.087)	0.092 (0.065–0.134)	1489	−3.270	0.001

Note: Data are presented as median (interquartile range). NAR was calculated as absolute neutrophil count (×10^9^ cells/L) divided by serum albumin (g/L); the values shown are untransformed. Abbreviations: CAR, creatinine-to-albumin ratio; IBI, inflammatory burden index; ICUAW, intensive care unit-acquired weakness; NAR, neutrophil-to-albumin ratio; SUCR, serum urea-to-creatinine ratio.

**Table 6:** Discriminatory performance and diagnostic characteristics of composite biomarkers.

Biomarker	AUC	95% CI	*p* Value	Youden Cutoff	Sensitivity, %	Specificity, %	Youden Index
NAR	0.645	0.550–0.740	0.004	0.386	43.9	82.1	0.259
IBI	0.602	0.506–0.697	0.044	388.226	82.5	41.0	0.235
CAR	0.637	0.538–0.736	0.007	4.267	57.9	80.8	0.387
SUCR	0.665	0.570–0.760	0.001	0.064	78.9	51.3	0.302

Note: The cutoffs, sensitivities, specificities, and Youden indices shown here are apparent estimates derived and evaluated in the same cohort. Bootstrap internal validation is reported in Supplementary Table S1; no external validation was performed. The NAR cutoff is reported on the untransformed ratio scale. Abbreviations: AUC, area under the receiver operating characteristic curve; CAR, creatinine-to-albumin ratio; CI, confidence interval; IBI, inflammatory burden index; NAR, neutrophil-to-albumin ratio; SUCR, serum urea-to-creatinine ratio.

## Data Availability

The datasets used and/or analyzed during the current study are not publicly available due to patient privacy and institutional restrictions but may be available from the corresponding author upon reasonable request and with appropriate institutional and ethics approval.

## Supplementary Materials

The following supporting information can be downloaded at https://www.mdpi.com/article/10.3390/jcm15208171/s1. Table S1. Bootstrap Internal Validation of Composite Biomarker Cutoffs.

## References

[1] Zhang Y., Wang Y., Yang J., Li Q., Zhou M., Lu J., Hu Q., Ma F. (2025). Development and validation of machine learning-based risk prediction models for ICU-acquired weakness: A prospective cohort study. Eur. J. Med. Res..

[2] De Noray H., Duclos N.C., Boyer A., Gallice T. (2025). Handgrip strength association with weaning outcome in mechanically ventilated ICU patients: A systematic review and meta-analysis. Crit. Care.

[3] Appleton R.T.D., Kinsella J., Quasim T. (2015). The incidence of intensive care unit-acquired weakness syndromes: A systematic review. J. Intensive Care Soc..

[4] Hermans G., Van Mechelen H., Clerckx B., Vanhullebusch T., Mesotten D., Wilmer A., Casaer M.P., Meersseman P., Debaveye Y., Van Cromphaut S. (2014). Acute outcomes and 1-year mortality of intensive care unit-acquired weakness: A cohort study and propensity-matched analysis. Am. J. Respir. Crit. Care Med..

[5] Oestreicher V., Hennemann M., Hilfiker R., Faivre T., Zoni G., Demont M., Teixeira M., Ucchino R., Tassaux D., Piquilloud L. (2025). Persistent inspiratory muscle weakness among extubated patients after prolonged intubation is frequent and can be predicted early by maximal inspiratory pressure measured 12 days after its initial diagnosis: A prospective observational study. Crit. Care.

[6] Ackerman M.H., Ahrens T., Kelly J., Pontillo A. (2021). Sepsis. Crit. Care Nurs. Clin. N. Am..

[7] Yamada K., Kitai T., Iwata K., Nishihara H., Ito T., Yokoyama R., Inagaki Y., Shimogai T., Honda A., Takahashi T. (2023). Predictive factors and clinical impact of ICU-acquired weakness on functional disability in mechanically ventilated patients with COVID-19. Heart Lung.

[8] Zhang X., Wang Z., Wang J., Wu F., Xia L., Shi S., Zhu M., Zhuang J. (2025). Risk factors, diagnostic challenges, and emerging therapeutic strategies for ICU-acquired weakness: A brief review. J. Multidiscip. Healthc..

[9] Wang L., Lyu H., Shen Y., Jin L., Sheng H. (2021). Establishment and validation of a risk prediction model for intensive care unit-acquired weakness. Zhonghua Wei Zhong Bing Ji Jiu Yi Xue.

[10] Fan E., Cheek F., Chlan L., Gosselink R., Hart N., Herridge M.S., Hopkins R.O., Hough C.L., Kress J.P., Latronico N. (2014). An official American Thoracic Society clinical practice guideline: The diagnosis of intensive care unit-acquired weakness in adults. Am. J. Respir. Crit. Care Med..

[11] Chen H., Li X., Zhou L., Qiang X. (2025). Research progress on ICU-acquired weakness in sepsis patients. Zhonghua Wei Zhong Bing Ji Jiu Yi Xue.

[12] Latronico N., Gosselink R. (2015). A guided approach to diagnose severe muscle weakness in the intensive care unit. Rev. Bras. Ter. Intensiv..

[13] Xie Y., Liu S., Zheng H., Cao L., Liu K., Li X. (2020). Utility of plasma GDF-15 for diagnosis and prognosis assessment of ICU-acquired weakness in mechanically ventilated patients: Prospective observational study. Biomed. Res. Int..

[14] Ridley E.J., Lambell K. (2022). Nutrition before, during and after critical illness. Curr. Opin. Crit. Care.

[15] Xie H., Yuan K., Ruan G., Wei L., Zhang H., Ge Y., Lin S., Song M., Wang Z., Liu C. (2023). Improving the assessment of malnutrition in cancer: Using systemic inflammation markers as a supplement to the inflammation items of the GLIM criteria. Clin. Nutr..

[16] Liu S., Zhang C., Feng Y. (2026). The relationship between serum creatinine-to-albumin ratio (CAR) and prognosis in critically ill patients with acute heart failure admitted to the ICU: A retrospective study based on the MIMIC-IV database. Medicine.

[17] Eggmann S., Parry S.M., Broadley T., Lynch G.S., Bongetti A.J., Ridley E.J., Ayre E., Bailey M., Bellomo R., Berney S. (2025). The intensive care medicine research agenda for the management of ICU acquired weakness: A multinational, interprofessional perspective. Intensive Care Med..

[18] Hillinger P., Levé A.M.-L., Woyke S., Ronzani M., Kreutziger J., Schmid S., Rugg C. (2025). The impact of protein feed on the urea-to-creatinine ratio: A retrospective single-center study. Nutrients.

[19] Wu Y., Ding N., Jiang B., Zhang Z., Zhang C., Li B. (2018). Diagnostic tools of intensive care unit acquired weakness: A systematic review. Zhonghua Wei Zhong Bing Ji Jiu Yi Xue.

[20] Benchimol E.I., Smeeth L., Guttmann A., Harron K., Moher D., Petersen I., Sørensen H.T., von Elm E., Langan S.M. (2015). The REporting of studies Conducted using Observational Routinely-collected health Data (RECORD) statement. PLoS Med..

[21] IBM Corp (2019). IBM SPSS Statistics for Windows.

[22] Engelhardt L.J., Grunow J.J., Wollersheim T., Carbon N.M., Balzer F., Spranger J., Weber-Carstens S. (2022). Sex-specific aspects of skeletal muscle metabolism in the clinical context of intensive care unit-acquired weakness. J. Clin. Med..

[23] Liu J., Xu Z., Luo S., Bai Y., Feng J., Li F. (2024). Risk factors for ICU-acquired weakness in sepsis patients: A retrospective study of 264 patients. Heliyon.

[24] Li T., Wang D., Xu L., Xu Z., Li Z., Bai X., Zhao H., Yang J., Liu Y., Wang Y. (2025). The role of heme oxygenase-1 (HO-1) in sepsis-associated organ damage: A systematic review. Pathol. Res. Pract..

[25] Xiong C., Guan F., Feng J., Jia J., Zhang J., Tu R., Li J., Zhou J., Wang J., Liu L. (2025). Prolonged sepsis triggers abnormal mitochondrial dynamics in the limb muscles and diaphragm. Mol. Cell Biochem..

[26] Michel-Flutot P., Vinit S., Mansart A. (2025). Pathophysiology of neuromuscular weakness acquired in intensive care during sepsis. Med. Sci..

[27] Asis A., McMahon D., Fujitani S., Povoa P., Rodriguez A., Reyes L.F., Martin-Loeches I. (2026). Severe community-acquired pneumonia: An integral approach. J. Intensive Care.

[28] Shao L., Liang Y. (2025). Predictive value of bedside lung ultrasound, quantitative chest CT, and frailty assessment for short-term outcomes in elderly patients with severe pneumonia: A pilot study. BMC Pulm. Med..

[29] Jin C., Hu F., Wu L., Zhang L., Wu B., Yang Y. (2025). Risk factors of ICU-acquired weakness in sepsis patients. Pak. J. Med. Sci..

[30] Weber-Carstens S., Deja M., Koch S., Spranger J., Bubser F., Wernecke K.D., Spies C.D., Spuler S., Keh D. (2010). Risk factors in critical illness myopathy during the early course of critical illness: A prospective observational study. Crit. Care.

[31] Mayer K.P., Teixeira J.P., González-Seguel F., Tran V.Q., Gross J.M., Horikawa-Strakovsky A., Pal C.A., Shareef Z.T., Israel H.P., Wen Y. (2025). Acute skeletal muscle wasting in patients with acute kidney injury requiring continuous kidney replacement therapy: A prospective multicenter study. J. Crit. Care.

[32] Patt H., Bandgar T., Lila A., Shah N. (2013). Management issues with exogenous steroid therapy. Indian. J. Endocrinol. Metab..

[33] Zhao M., Fan Y., Wu T., Yu L., Li H., Zeng H., Wu Y. (2025). Risk factors for ICU-acquired weakness in patients undergoing mechanical ventilation: A systematic review and meta-analysis. J. Thorac. Dis..

[34] Hermann B., Decormeille G., Gobé T., Mangeard N., Maamar A., Sayadi S., Pernod B., Robquin N., Ponthus J.-P., Le Potier S. (2025). Neuromuscular blockade and their monitoring in the intensive care unit: A multicenter observational prospective study. Ann. Intensive Care.

[35] Reid M.B., Judge A.R., Bodine S.C. (2014). CrossTalk opposing view: The dominant mechanism causing disuse muscle atrophy is proteolysis. J. Physiol..

[36] Evans D.C., Corkins M.R., Malone A., Miller S., Mogensen K.M., Guenter P., Jensen G.L. (2021). the ASPEN Malnutrition Committee. The use of visceral proteins as nutrition markers: An ASPEN position paper. Nutr. Clin. Pract..

[37] Zhang Y., Chen X., Gu Y., He X., Zhang H., Sun L., Zhang Y., Liu B., Wang X., Ma Y. (2026). The neutrophil-to-albumin ratio is associated with in-hospital mortality in patients with cirrhosis and pneumonia: A multicenter retrospective cohort study. BMC Infect. Dis..

[38] Xie H., Ruan G., Ge Y., Zhang Q., Zhang H., Lin S., Song M., Zhang X., Liu X., Li X. (2022). Inflammatory burden as a prognostic biomarker for cancer. Clin. Nutr..

[39] Xie H., Ruan G., Wei L., Deng L., Zhang Q., Ge Y., Song M., Zhang X., Lin S., Liu X. (2023). The inflammatory burden index is a superior systemic inflammation biomarker for the prognosis of non-small cell lung cancer. J. Cachexia Sarcopenia Muscle.

[40] Xie X., Liu Y., Zhi S., Huang W., Li N. (2025). Effect of octreotide combined with ulinastatin on the creatinine-to-albumin ratio and systemic immune-inflammation index in patients with severe acute pancreatitis. Am. J. Transl. Res..

[41] Haines R.W., Zolfaghari P., Wan Y., Pearse R.M., Puthucheary Z., Prowle J.R. (2019). Elevated urea-to-creatinine ratio provides a biochemical signature of muscle catabolism and persistent critical illness after major trauma. Intensive Care Med..

