# Supplementary material for: Clinical Factors and Composite Biomarkers Associated with Intensive Care Unit-Acquired Weakness: A Retrospective Cohort Study
Source: J Clin Med. 2026 Oct 21;15(20):8171. doi: 10.3390/jcm15208171 (PMC13706466; doi:10.3390/jcm15208171)
Supplement: Supplementary file 1 [file jcm-15-08171-s001.zip › jcm-4588508-supplementary.pdf]

Supplementary Table S1 Bootstrap Internal Validation of Composite Biomarker Cutoffs

Biomarker	Full-cohort cutoff	Bootstrap cutoff median (2.5th–97.5th)	In-bag AUC median (2.5th–97.5th)	Out-of-bag AUC mean (2.5th–97.5th)	Out-of-bag sensitivity, % mean (2.5th–97.5th)	Out-of-bag specificity, % mean (2.5th–97.5th)	Out-of-bag Youden index, mean
**NAR**	0.386	0.354 (0.182–0.462)	0.646 (0.544–0.741)	0.645 (0.505–0.776)	50.0 (20.0–88.2)	66.6 (23.1–92.9)	0.166
**IBI**	388.226	388.226 (137.404–2293.747)	0.603 (0.498–0.697)	0.599 (0.462–0.727)	72.8 (25.0–95.0)	41.2 (18.5–76.5)	0.140
**CAR**	4.267	4.267 (3.377–4.450)	0.639 (0.543–0.742)	0.635 (0.513–0.767)	56.0 (38.1–73.7)	78.7 (62.1–90.3)	0.347
**SUCR**	0.064	0.068 (0.058–0.115)	0.664 (0.566–0.756)	0.666 (0.539–0.791)	62.9 (26.3–89.5)	58.0 (31.0–87.0)	0.209

Notes: The analysis included 135 patients (57 with ICUAW and 78 without ICUAW). For each of 1,000 stratified patient-level bootstrap resamples, patients were sampled with replacement separately within each outcome group to preserve group sizes. The cutoff maximizing the Youden index was selected in each resample; the lowest cutoff was selected if several cutoffs tied. Each cutoff was then applied to patients not selected in that resample (out-of-bag) to estimate sensitivity and specificity. Higher biomarker values were classified as ICUAW. AUC was calculated from continuous biomarker values independently of the selected cutoff. Random seed: 20261002.

Values in parentheses are empirical 2.5th–97.5th percentiles across the 1,000 valid resamples. Bootstrap cutoffs and in-bag AUCs are summarized by medians; out-of-bag metrics are summarized by means. These percentile ranges describe internal resampling variability and are not external-validation confidence intervals. Full-cohort cutoffs are provided for comparison with Table 6.

Cutoffs retain the numerical scales used in the main manuscript; NAR is expressed in 10^9 cells/g. No additional transformation or rescaling was applied for this bootstrap analysis.

Abbreviations: AUC, area under the receiver operating characteristic curve; CAR, creatinine-to-albumin ratio; IBI, inflammatory burden index; NAR, neutrophil-to-albumin ratio; SUCR, serum urea-to-creatinine ratio.
